# Human Amniotic Fluid Mesenchymal Stem Cells Improve Ovarian Function During Physiological Aging by Resisting DNA Damage

**DOI:** 10.3389/fphar.2020.00272

**Published:** 2020-03-26

**Authors:** Boxian Huang, Chenyue Ding, Qinyan Zou, Jiafeng Lu, Wei Wang, Hong Li

**Affiliations:** ^1^Center of Reproduction and Genetics, Affiliated Suzhou Hospital of Nanjing Medical University, Suzhou Municipal Hospital, Suzhou, China; ^2^State Key Laboratory of Reproductive Medicine, Nanjing Medical University, Nanjing, China

**Keywords:** ovarian physiologic aging, human amniotic fluid mesenchymal stem cells, DNA damage, oogenesis, anti-apoptosis

## Abstract

Many studies have shown that mesenchymal stem cells have the ability to restore function in models of premature ovarian insufficiency disease, but few studies have used stem cells in the treatment of ovarian physiologic aging (OPA). This experimental study was designed to determine whether human amniotic fluid mesenchymal stem cells (hAFMSCs) have the ability to recover ovarian vitality and to determine how they function in this process. Mice (12–14 months old) were used in this study, and young fertile female mice (3–5 months old) were the control group. Ovarian markers for four stages of folliculogenesis and DNA damage genes were tested by qPCR and western blot. hAFMSCs were used to treat an OPA mouse model, and the animals treated with hAFMSCs displayed better therapeutic activity in terms of the function of the mouse ovary, increasing follicle numbers and improving hormone levels. In addition, our results demonstrated that the marker expression level in ovarian granular cells from patients with OPA was elevated significantly after hAFMSC treatment. In addition, the proliferation activity was improved, and apoptosis was dramatically inhibited after hAFMSCs were cocultured with hGCs from OPA patients. Finally, in this study, hAFMSCs were shown to increase the mRNA and protein expression levels of ovarian markers at four stages of folliculogenesis and to inhibit the expression of DNA damage genes. These works have provided insight into the view that hAFMSCs play an integral role in resisting OPA. Moreover, our present study demonstrates that hAMSCs recover ovarian function in OPA by restoring the expression of DNA damage genes.

## Introduction

Ovarian physiologic aging (OPA) is a worldwide reproductive health problem (White et al., 2012). A gradual decrease in both the quantity of oocytes in the ovarian cortex could identify the ovarian aging procedure, especially after 40 years of age (Jiao et al., 2018). Menopause is an inevitable stage during ovarian aging (Herbert et al., 2015). The growing primordial follicle pool serves as the initial source of developing follicles during stages of meiosis, but it mirrors the lifetime of ovaries in mice, suffering an inevitable decline with increasing age (Ding et al., 2018a).

Human granulosa cells (hGCs) are a significant class of ovarian cells. They are situated outside of the zona pellucida in oocytes and are connected to oocytes *via* gap junctions (Ding et al., 2017). They play a crucial role in the maturation process inside and outside of follicle responsiveness. Current studies have indicated that, in the normal developmental period of the ovarian follicular cycle, all phases of follicular atresia are closely related to the activity levels of granulosa cells (Sun et al., 2012). Moreover, apoptosis in granulosa cells is the principal mechanism driving follicular atresia and arrest (Ding et al., 2018b). It has been speculated that abnormal folliculogenesis is probably due to increased apoptosis and decreased proliferation of granulosa cells (Ding et al., 2018c). The destiny of ovarian follicles might determine the equilibrium between cell survival and cell death signaling pathways in granulosa cells, which are regulated by many genes, such as FSHR, AMH, FOXL2, and CYP19A1 (Huang et al., 2018).

Persistent senescent cells are generated by different situations related to human health throughout organismal life: age-related disease, natural aging, and therapeutic intervention-induced aging (Childs et al., 2015). DNA damage is a particularly problematic issue for nondividing or slow cell cleavage, where unrepaired damage can cumulate over time (Goldmann et al., 2018). In proliferating hGCs, an uncapped free double-stranded chromosome end is ultimately exposed by progressive telomere erosion and triggers a permanent DNA damage response (DDR) (Marangos et al., 2015). To address DNA damage, the DDR has evolved with cells and is an intricate and delicately regulated genomic maintenance apparatus that senses and signals the presence of lesions to finally promote their repair (Vandormael-Pournin et al., 2015). The DDR triggers distinct repair pathways and is regulated by specific genes, including γH2AX, BRCA1, PARP1, and XRCC6; each is specific to a particular category of DNA dysfunction and aims at restoring the integrity of the damaged double-stranded DNA molecule (Lane et al., 2017).

The therapeutic activity of human amniotic fluid mesenchymal stem cells (hAFMSCs) has been demonstrated in preclinical investigations. Previous researchers have used many types of MSCs to repair damaged ovaries in rats or act as therapeutic agents to treat ovarian insufficiency disease, such as human amniotic MSCs, human adipose MSCs, and human umbilical cord MSCs (Bossolasco et al., 2006; Carraro et al., 2008). However, few studies have revealed whether hAFMSCs can prevent OPA. MSCs have the capability to reduce tissue damage, prevent fibrotic remodeling and apoptosis, recover cellular function, promote angiogenesis, stimulate endogenous stem cell maintenance and differentiation, and even diminish the immune response (Wang et al., 2014). hAFMSCs have been shown to be a new valuable source of stem cells in the perinatal period and could be used for cell-based treatment. hAFMSCs exhibit several advantages and are suitable for clinical application; they are acquired from plentiful tissue sources, are poorly immunogenic, and are stably proliferative, while their non-injurious nature provides them with considerable ability to perform repair and regeneration of autologous cells (Baksh et al., 2004).

Nevertheless, there is little known regarding whether hAFMSCs contain the potential to oppose OPA and how they might perform this function. Therefore, the aim of the current study was to determine whether hAFMSCs have the power to restore normal ovarian function in OPA disease.

## Materials and Methods

### Preparation and Culture of hAFMSCs

All samples of amniotic fluid cells were collected *via* amniocentesis that were performed under sterile conditions, and the residual fluid from prenatal diagnoses was used for research. Amniotic fluid samples with abnormal karyotypes were excluded. The range of maternal age was between 30–35 years old, and the range of gestational age was between 15–22 weeks (healthy pregnancy). Follicular fluid samples (2–5 ml from every sample) were collected separately. This study was executed with the approval of the Ethics Committee of Suzhou Hospital, and informed consent was acquired from each participant. To obtain hAFMSCs, we centrifuged amniotic fluid samples at 3000 × g for 5 min and discarded the supernatant. Then, hAFMSCs were seeded and cultured in DMEM/F12 medium (Gibco, USA) supplemented with 1% glutamine, 1% penicillin/streptomycin (Gibco, USA), 10% FBS (Gibco, USA), and bFGF (2 ng/ml, Invitrogen, USA) at 37°C in a 5% CO_2_ atmosphere. After reaching 70% to 80% confluence, cells were passaged following incubation in 0.25% trypsin (Thermo Fisher Scientific, USA) at 37°C for 5 min. Approximately 3×10^7^ cells were obtained, and the cells were plated on a 10 cm cell culture dish. hAFMSCs at the fourth–eighth passage were tested.

### The Phenotypic Characterization of hAFMSCs

The expression of CD34, CD105, CD73, CD90, and CD29 was tested to characterize the phenotype of hAFMSCs by means of FACS. The exact method used was the same as the FACS analysis described elsewhere in the methods section. Differentiation kits were used to test the ability of hAFMSCs to differentiate (Thermo, USA).

### Isolation of Primary Human Ovarian Granulosa Cells (hGCs) From Individuals With OPA

As described in a previous study of ours (Ding et al., 2017), young patients (under 40 years) with tubal occlusion were established as a control group. The inclusion criteria for the selection of individuals with OPA included women older than 40 years with an AMH < 1.1 ng/ml, FSH ≥ 10 mIU/mL and antral follicle count < 5. Women with known abnormal karyotypes, ovarian surgery, or previous autoimmune diseases were excluded. hGCs were primarily cultured in six-well plates containing DMEM/F12 medium (Thermo, USA) supplemented with 100 mg/ml streptomycin sulfate (Thermo, USA), 10% FBS (complete medium), 1% penicillin/streptomycin, and 1× GlutaMAX (Thermo, USA). In all experiments, cells were fed every two days.

### Establishment of an OPA Mouse Model

Ovarian physiologic aging female C57BL/6J mice (12–14 months old, SPF class, from the Jackson Lab) and young, fertile female C57BL/6J mice (3–5 months old, SPF class) were purchased from Nanjing Medical University. All experiments were approved by the Institutional Animal Care and Use Committee and were conducted following institutional guidelines. The mice were bred in conditions with a 12-hour light/dark cycle at a temperature of 28 ± 2°C. To determine the estrous cycle, mouse vaginal smears were checked at 9:00 am every day. Only young mice with normal estrous cycles and OPA mice with disorganized estrous cycles were used in this study. Aging mice were randomly distributed into the control group (young mice) and treatment group (n = 15 per group).

### Coculture of hGCs With hAFMSCs and Injection of hAFMSCs Into OPA Mouse Ovaries

hGCs were divided into three groups, namely, the OPA hGCs group, the hAFMSCs cocultured with OPA hGCs group, and the young hGCs group. Tests were performed 7 days after culturing the hGCs with hAFMSCs. There were three mouse groups: the hAFMSC-injected OPA mouse model group, the OPA mouse model group, and the young mouse group. A total of 4 weeks after the hAFMSCs were injected into the ovary with OPA, hematoxylin and eosin (HE) experiments were conducted to calculate the follicle number.

### Calculation of Ovarian Follicle Counts

The mice (with or without transplanted cells) were euthanized between 0 and 4 weeks after hAFMSC treatment. Ovaries from both sides of each mouse were extracted and fixed in 10% formalin. Then, ovaries were paraffin embedded, serially sectioned to produce 5 μm thick sections, and stained with HE. Primary, primordial, secondary, and antral follicles were classified, and their numbers were calculated by choosing three typical sections from each ovary. Only follicles containing an oocyte were counted to avoid calculating a follicle twice. All experiments were conducted three times. The results are presented as the mean ± standard deviation, and *p* < 0.05 was considered statistically significant.

### Flow Cytometry (FACS) Analysis

hAFMSCs or hGCs were digested separately using 0.25% trypsin-EDTA for 5 min before being mixed well to produce a suspension of single cells. A Cytofix/Cytoperm Fixation/Permeabilization Solution kit (BD, USA) was used to permeabilize and fix the samples according to the manufacturer's instructions. PE- or FITC-conjugated antibodies or their relevant isotype controls were used to characterize hAFMSCs and hGCs. Finally, cells were analyzed using a fluorescence-activated cell sorter (Beckman, USA). Information concerning the primary antibodies used is provided in **Supplementary Table 2**. All tests were performed at least three times. The results are presented as the mean ± standard deviation, and *p* < 0.05 was considered statistically significant.

### Enzyme-Linked Immunosorbent Assay (ELISA) Analysis

The levels of FSH, E2, or AMH in the plasma of the OPA mouse model were evaluated with an ELISA kit (MyBioSource, USA) following the manufacturer's instructions. In brief, 50 μl of serum sample was added to each well of the test plate before being sealed with a membrane and incubated at 37°C for 30 min. Then, the wells of the plate were dried and washed with wash buffer five times. Subsequently, 50 μl of an HRP-conjugated reagent was added to each well of the plate, and then the plate was incubated at 37°C for 60 min. The plate was washed again with wash buffer five times. Afterwards, each well received 50 μl of substrate A solution and 50 μl of substrate B solution before being incubated at 37°C for 15 min. Finally, 50 μl of stop solution was added to stop the reaction, and the plate was scanned with a spectrophotometer (Varian Company, Australia) to measure and record the value of light absorbance.

### Western Blot Analysis

hGCs and ovaries were gathered and dissolved in lysis buffer as previously discussed. Total protein was extracted and loaded on 10% gels. Then, the protein was separated by SDS-PAGE (sodium dodecyl sulfate-polyacrylamide gel electrophoresis), and the proteins were then electrotransferred to polyvinylidene difluoride membranes (PVDF, Millipore, USA). The membranes were incubated overnight at 4°C with primary antibodies. Then, appropriate secondary antibodies (goat anti-rabbit HRP conjugates; Jackson Immuno Research, West Grove, USA) were incubated separately with the membranes. After washing three times with Tris-buffered saline, a chemiluminescence kit was used to enhance specific signals (Pierce ECL Western Blotting Substrate, Thermo). Finally, the membrane was examined with an imaging detection system (Tanon, China), and ImageJ software (National Institutes of Health, USA) was used to analyze immunoreactive bands. All experiments were repeated at least three times. The results are presented as the mean ± standard deviation, and *p* < 0.05 was considered statistically significant. Information regarding the primary antibodies used for western blot analyses is listed in **Supplementary Table 3**.

### Statistical Analysis

All results are shown as the means ± standard deviation. SPSS 17.0 and GraphPad Prism 8 software were used in this study, and *p* < 0.05 was considered statistically significant. * *p* < 0.05, ** *p* < 0.01, and ***p < 0.001. Primer Premier 5.0 was used to design the primers for qPCR. The primer sequences are listed in **Supplementary Table 1**.

## Results

### Ovarian Function Was Restored by hAFMSCs in an OPA Mouse Model

**Figure 1:**
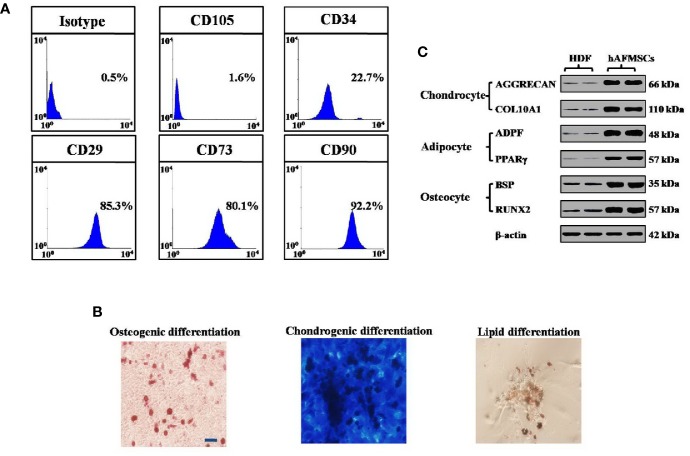
Characterization of hAFMSCs was performed. **(A)** The expression levels of CD105, CD29, CD34, CD73, CD90, and CD73 in hAFMSCs were determined by flow cytometry. **(B)** hAFMSCs can differentiate into adipocytes (oil red), osteoblasts (alizarin red), and chondroblasts (alcian blue) under standard *in vitro* differentiating conditions. **(C)** The protein expressions of osteogenic (BSP, RUNX2), adipogenic (ADPF, PPARƳ), and chondrogenic (AGGRECAN, COL10A1) cells were tested in hAFMSCs and in HDF (human dermal fibroblast cell line). Scale bars = 10 μm. hAFMSCs = human amniotic fluid mesenchymal stem cells.

**Figure 2:**
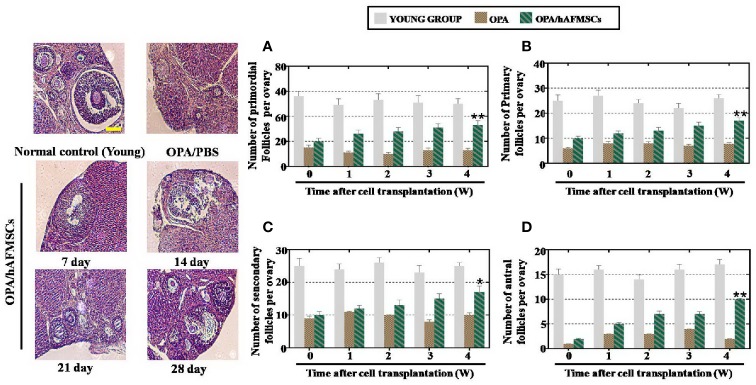
hAFMSCs improved the function of an OPA mouse model. **(A)** The number of primordial follicles was higher in the OPA/hAFMSC group than it was in the OPA group at 4 weeks. **(B)** The number of primary follicles was higher in the OPA/hAFMSC group than it was in the OPA group at 4 weeks. **(C)** The number of secondary follicles was higher in the OPA/hAFMSC group than it was in the OPA group at 4 weeks. **(D)** The number of antral follicles was higher in the OPA/hAFMSC group than it was in the OPA group at 4 weeks. The error bars indicate the SD; *p < 0.05; **, *p* < 0.01; (compared with the OPA group).

**Figure 3:**
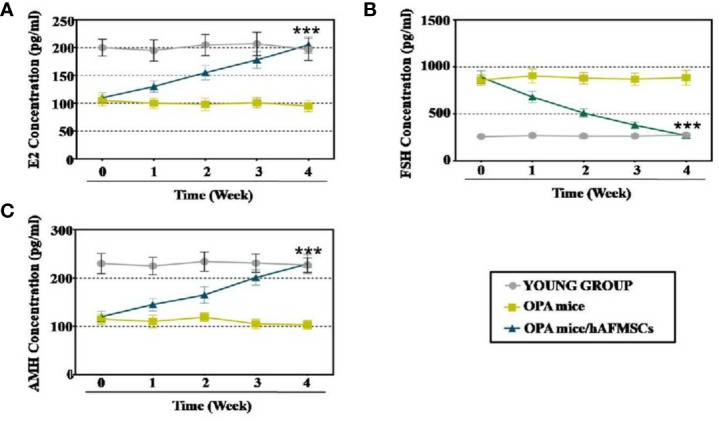
hAFMSCs improved hormone levels in the OPA mouse model. **(A)** The level of E2 was measured by ELISA over 4 weeks after hAFMSC transplantation. **(B)** The levels of the hormone FSH were measured by ELISA over 4 weeks after hAFMSC transplantation. **(C)** The levels of the hormone AMH were measured by ELISA over 4 weeks after hAFMSC transplantation. The error bars indicate the SD; ***, *p* < 0.001; (compared with the OPA group).

First, to evaluate the therapeutic outcomes of hAFMSCs in OPA disease, FACS was used to characterize the hAFMSCs. The results revealed that CD29, CD90, and CD73 were highly expressed, while the levels of other markers, CD105 and CD34, were reduced to a minimum in hAFMSCs (**Figure 1A**). hAFMSCs are multipotent mesenchymal stem cells that possess the capacity to differentiate into osteoblasts, adipocytes, and chondroblasts, as shown in **Figure 1B**. In addition, in **Figure 1C**, our results showed that the protein expressions of osteogenic (BSP, RUNX2), adipogenic (ADPF, PPARƳ), and chondrogenic (AGGRECAN, COL10A1) cells were higher in hAFMSCs than in HDF (human dermal fibroblast cell line). After ovaries of the OPA mice were injected with hAFMSCs, HE staining was performed, and it was proved that hAFMSCs could remarkably restore the follicle number of primordial follicles, primary follicles, secondary follicles, and antral follicles to 66%, 65%, 68%, and 59%, respectively, at week 4 in comparison with the control group (**Figures 2A–D**). Furthermore, we also tested the activity of hAFMSCs after injection. In our results, the survival time of hAFMSCs in OPA ovaries was over 7 days. After injection at 14 days, an immunofluorescence assay revealed that few hAFMSCs could be found in OPA ovaries (**Supplementary Figure 1**). After injection of hAFMSCs, the hormone level was assessed. In the therapy group, ELISA results demonstrated that the levels of AMH (64%) and E2 (67%) were slightly rescued compared with those of the OPA group (49% AMH and 51% E2) during 1 week (**Figures 3A, C**). However, after four weeks, the levels of AMH (101%) and E2 (99%) were rescued by the hAFMSCs, as shown in **Figures 3A, C**. In addition, the level of FSH in the treatment group decreased to 251%, which was less than that of the OPA group (334%) during 1 week. In **Figure 3B**, a comparison after 4 weeks of treatment with hAFMSCs shows that the FSH level recovered to normal levels (101%) and was significantly different from that of the control group.

Overall, hAFMSCs demonstrated a potent ability to regenerate ovarian function in OPA disease.

### hAFMSCs Increased the Marker Expression Levels of OPA-hGCs

**Figure 4:**
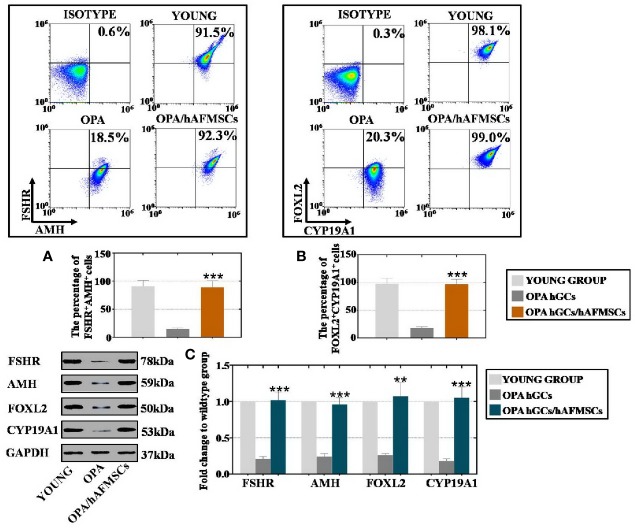
hAFMSCs upregulated the expression level of hGC markers in OPA disease, **(A)** hAFMSCs increased the number of FSHR^+^AMH^+^ positive hGCs. **(B)** hAFMSCs increased the number of FOXL2^+^CYP19A1^+^ positive hGCs. **(C)** hAFMSCs increased the protein level of hGC markers (FSHR, AMH, FOXL2, and CYP19A1). The error bars indicate the SD; **, *p* < 0.01; ***, *p* < 0.001; (compared with the OPA group).

To examine the therapeutic outcome of hAFMSCs on patients with OPA in the clinic, hGCs were collected from the young and OPA groups to analyze the effects of marker expression after coculture with hAFMSCs; these experiments were performed in our reproductive center as previously reported (Ding et al., 2017). FACS and western blotting were used to calculate the impacts of hAFMSCs on hGC markers, such as FOXL2 (follicular activation), CYP19A1 (ovary formation), AMH (follicular growth), and FSHR (follicular maturation). The FACS results revealed that coculture with hAFMSCs resulted in a greater increase (92%) in the number of FSHR^+^AMH^+^ cells than what was observed in the OPA group (18%), as shown in **Figure 4A**. As shown in **Figure 4B**, the FACS assay results demonstrate that hAFMSCs resulted in a higher increase (99%) in the number of FOXL2^+^CYP19A1^+^ cells than what was observed in the OPA group (20%). Western blot experiments indicated that hAFMSCs produced a more dramatic increase in the expression of FSHR (102%), AMH (96%), FOXL2 (107%), and CYP19A1 (105%) than the OPA group did (21%, 24%, 26%, and 18%, respectively), as shown in **Figure 4C**.

In summary, powerful recovery effects were exhibited by hAFMSCs for hGCs with OPA.

### hAFMSCs Enhanced the Proliferation Rate and Suppressed the Apoptosis Level of hGCs With OPA

**Figure 5:**
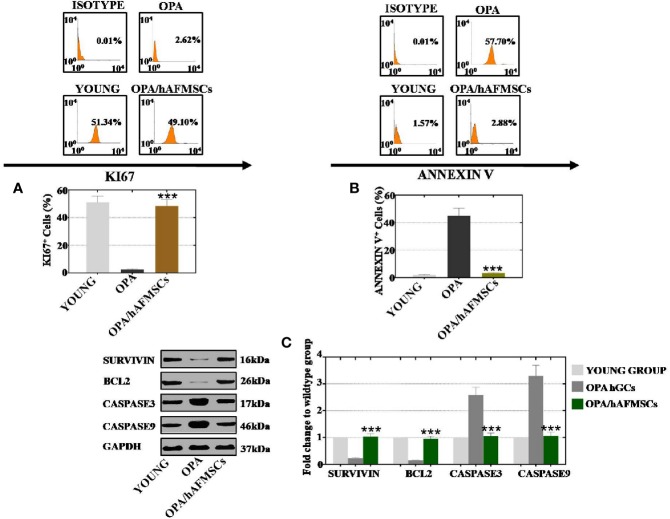
hAFMSCs improved proliferation and inhibited the apoptosis level to the normal level after coculture with OPA hGCs. **(A)** hAFMSCs improved proliferation to the normal level in hGCs with OPA. **(B)** hAFMSCs inhibited apoptosis, so that the rate returned to a normal level in hGCs with OPA. **(C)** hAFMSCs increased the expression of the anti-apoptosis genes (SURVIVIN and BCL2) and decreased the expression of the apoptosis genes (CASPASE 3 and CASPASE 9) to normal levels. The error bars indicate the SD; ***, *p* < 0.001; (compared with the OPA group).

To examine the effects of hAFMSCs on apoptosis and cell proliferation, hGCs and hAFMSCs were cultured together for 7 days. A FACS assay was applied to evaluate cell activity. As shown in **Figure 5A**, the proliferation level of hGCs in the hAFMSC group was increased to 49%, and these rates were greater than those of the OPA group (3%) and were comparable to those of the young group (51%). The results of the FACS assay similarly implied that the rate of apoptosis was decreased to 3% in the hAFMSC group, and these rates were less than that in the OPA group (57%) and were comparable to those of the young group (1.6%), as shown in **Figure 5B**. Additionally, apoptosis genes (CASPASE 3 and CASPASE 9) and apoptosis resistance genes (BCL2 and SURVIVIN) were analyzed at the protein level. After hAFMSCs were cocultured with OPA-hGCs, hAFMSCs exhibited increased expression levels of SURVIVIN (103%) and BCL2 (96%)—more than what was observed in the OPA group (23% and 15%) in **Figure 5C**. Furthermore, western blot results also demonstrated that hAFMSCs caused a decrease in the expression levels of CASPASE 3 (105%) and CASPASE 9 (106%) that was greater than those in the OPA group (259% and 330%, respectively) when compared to the young group (**Figure 5C**).

In conclusion, hAFMSCs could enhance proliferation and prevent apoptosis of hGCs with OPA.

### hAFMSCs Recovered the Marker Expression Levels of OPA Ovaries in Different Stages

**Figure 6:**
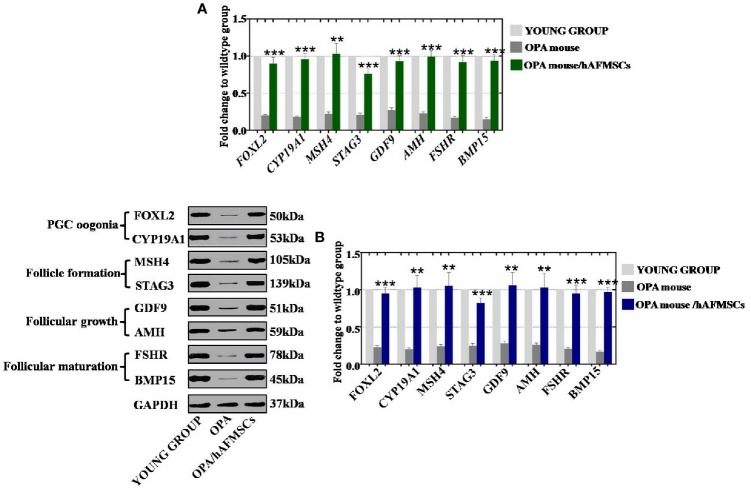
hAFMSCs elevated ovary expression after injection into an OPA mouse model. **(A)** qPCR assays showed that hAFMSCs increased ovarian marker expression at four stages: PGC oogonia (FOXL2 and CYP19A1), follicle formation (MSH4 and STAG3), follicular growth (GDF9 and AMH), and follicular maturation (FSHR and BMP15). **(B)** Western blot assay showed that hAFMSCs increased ovarian marker expression at the four stages. The error bars indicate the SD; **, p < 0.01; ***, p < 0.001.

To determine how the ovarian function of the OPA mouse model was enhanced by therapy with hAFMSCs, hAFMSCs were injected into the mouse OPA model ovary for 1 month. The follicular regulation of gene expression levels at different stages was tested by qPCR and western blot methods, and it included the assessment of the following genes and stages: PGC oogonia stage (FOXL2 and CYP19A1), follicle formation stage (MSH4 and STAG3), follicular growth stage (GDF9 and AMH), and follicular maturation stage (FSHR and BMP15). The mRNA assay results demonstrated that hAFMSCs decreased the expression of the PGC (primordial germ cells) and oogonia markers (FOXL2 and CYP19A1) to 90% and 96%, respectively, which was a more dramatic effect than that observed in the OPA group (20% and 18%, respectively) when compared to the levels in the young group (**Figure 6A**). The expression of follicle formation markers (MSH4 and STAG3) exhibited greater increases, to 103% and 76%, following hAFMSCs injection than the levels observed in the OPA group (22% and 21%), and these are all seen in comparison to those of the young group in **Figure 6A**. Moreover, in the hAFMSC treatment group, the expression levels of markers in the follicular growth stage were significantly increased to 93% and 99% for GDF9 and AMH, respectively, in the OPA group (27% and 23%) compared with those in the control group (**Figure 6A**). The expression of FSHR and BMP15 in the follicular maturation stage was significantly increased to 92% and 94% in the hAFMSC treatment group, respectively, which was a more powerful increase than that of the OPA group (17% and 15%, respectively); all of these changes are relative to the control group (**Figure 6A**). Moreover, the results of the protein assay showed that hAFMSCs increased the expression of PGC oogonia markers (FOXL2 and CYP19A1) to 95% and 103%, respectively, which was a greater change than the OPA group (23% and 20%); percentages are relative to that of the young group (**Figure 6B**). After therapeutic treatment with EGF, the expression of follicle formation markers (MSH4 and STAG3) was significantly elevated to the normal level (105% and 82%, respectively)—higher than they were in the OPA group (24% and 25%); percentages are relative to those of the control group in our western blot assay results (**Figure 6B**). The expression of follicular growth stage markers (GDF9 and AMH) exhibited greater increases, to 106% and 103%, by hAFMSCs treatment than did the OPA group (28% and 26%, respectively); this is all relative to the changes marked in the young group (**Figure 6B**). The expression of FSHR and BMP15 in the follicular maturation stage was significantly increased to 95% and 97%, respectively, in the hAFMSC treatment group, which was a greater increase than what was seen in the OPA group (21% and 17%, respectively); this is all relative to the levels of the control group (**Figure 6B**).

In total, hAFMSCs recovered marker expression at different stages.

### hAFMSCs Resisted DNA Damage in hGCs and Ovaries With OPA

**Figure 7:**
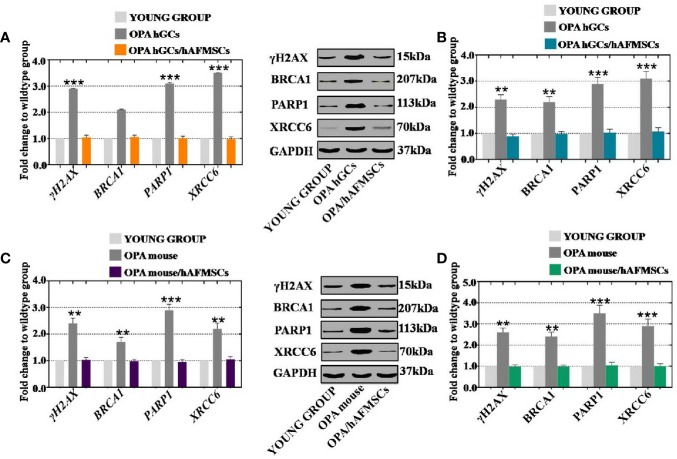
hAFMSCs increase the DNA damage genes *in vitro* and *in vivo*. **(A)** qPCR method revealed that hAFMSCs upregulated the DNA damage genes (γH2AX, BRCA1, PARP1, and XRCC6) to the normal level after co-cultured with OPA hGCs; **(B)** Western blot method revealed that hAFMSCs upregulated the DNA damage genes to the normal level after co-cultured with OPA hGCs; **(C)** qPCR assay revealed that hAFMSCs upregulated the DNA damage genes to the normal level after an injection into an OPA mice model; **(D)** western blot assay revealed that hAFMSCs upregulated the DNA damage genes to the normal level after an injection into an OPA mice model. All of the experiments were carried out three times; the error bars indicate the SD; **p < 0.01; ***p < 0.001 (compared with the OPA group).

To determine how the vitality of OPA cells and ovaries were ameliorated by treatment with hAFMSCs, OPA hGCs and hAFMSCs were cultured together for 7 days, and hAFMSCs were injected into the OPA mouse model for 4 weeks. In a previous study, BRCA1, PARP1, and XRCC6 were found to be pivotal genes related to safeguarding DNA damage repair and maintaining integrity. γH2AX detection changed the temporal and spatial distribution of DSB formation. In our *in vitro* results, the qPCR assay results illustrated that hAFMSCs increased the expression levels of γH2AX (65%), BRCA1 (70%), PARP1 (90%), and XRCC6 (105%) to levels that were higher than those in the OPA group (23%, 34%, 36%, and 17%, respectively), relative to those of the young group (**Figure 7A**). Furthermore, the protein expression levels of γH2AX (75%), BRCA1 (80%), PARP1 (98%), and XRCC6 (95%) exhibited greater elevation following treatment by hAFMSCs than they did in the OPA group (41%, 28%, 32%, and 22%, respectively); all values are relative to those of the young group (**Figure 7B**). In vivo results showed that, after treating the OPA mouse model with hAFMSCs, the mRNA expression levels of γH2AX, BRCA1, PARP1, and XRCC6 were significantly elevated to levels that were 60%, 65%, 78%, and 95%, respectively, which are higher than those in the OPA group (18%, 30%, 32%, and 15%, respectively); all percentages are relative to those in the young group (**Figure 7C**). In addition, a protein test demonstrated that the expression levels of γH2AX, BRCA1, PARP1, and XRCC6 were significantly increased to 88%, 85%, 98%, and 101%, respectively, which were more dramatic increases than those seen in the OPA group (18%, 25%, 34%, and 29%, respectively); percentages are relative to those of the control group (**Figure 7D**).

## Discussion

Age-induced ovarian function decline is associated with infertility, miscarriages, aneuploidy, and birth defects, and such consequences generate significant medical and quality of life complications (Perheentupa and Huhtaniemi, 2009). A previous study indicated that chemotherapy causes damage to the DNA double-stranded and ovarian germ cell niche (Tatone and Amicarelli, 2013), resulting in significant loss of ovarian reserve and the failure of the ovary to recreate new oocytes (Nelson et al., 2013). In addition, previous work on the role of the ovarian microenvironment in oocyte reconstruction indicated that external factors could activate primordial follicles recruitment to reinstate oocyte function during ovarian aging (Ye et al., 2017). Therefore, a challenging task is to identify the regenerative signaling pathway for female mice with OPA.

In the present study, hAFMSC transplantation restored follicle numbers to normal levels at nearly four stages in the OPA mouse model (**Figure 2**). In the meantime, the hormone indices of E2, AMH, and FSH in serum were detected, and all of these were recovered to control group levels (**Figure 3**). To transition from bench to bedside, hGCs derived from OPA patients were cocultured with hAFMSCs. This report indicated that hAFMSCs elevated the marker expression of hGCs to normal levels (**Figure 4**). Our investigation therefore first verified that hAFMSCs have the potential to resist OPA. Furthermore, in the present study, survival time of hAFMSCs was limited after transplantation into the ovary (**Supplementary Figure 1**). Previous studies have indicated that immunosuppressive drugs prolonged the survival of human embryonic stem cells, which appeared to be the result of beneficiary effects of allotransplantation (Swijnenburg et al., 2008). Besides, the additional effect of rapamycin on MSCs was able to ameliorate allograft rejection in the brain (Togha et al., 2017). Therefore, immunosuppressants associated with hAFMSCs were likely to become a good choice for elevating transplantation efficiency in OPA disease.

Earlier research has shown that prolonged chemotherapeutic treatment could induce suppression of the proliferation pathway and promote apoptosis in growing oocytes in female mice (Ding et al., 2018). Our findings indicated that hAFMSCs augmented the level of proliferation and repressed apoptosis in OPA hGCs to normal levels (**Figure 5**). Furthermore, qPCR and western blot results directly demonstrated that hAFMSCs recovered the expression levels of markers in the ovary at different developmental stages to regular levels in the OPA mouse model (**Figure 6**). Our study has provided the first confirmation that hAFMSCs have the ability to withstand OPA. In addition, a previous study showed that the process of folliculogenesis included four stages: PGC oogonia, follicle formation, follicular growth, and follicular maturation. Our results revealed that, after hAFMSCs were injected into the OPA mouse model, marker expression was elevated at four developmental stages to normal levels (**Figure 6**).

Ovaries with mutations in genes are associated with increased DNA damage in primordial follicle oocytes (Marangos and Carroll, 2012). It is important to maintain the integrity of DNA in oocytes, which is essential for fertility preservation. DNA damage is a constant threat to cells, and its harmful effects accumulate with age. Moreover, earlier research found that the accumulation of DNA double-strand breaks, as represented by γH2AX protein expression, was accelerated in the primordial follicles of oocytes from women with gene mutations (Keogh et al., 2006). It seems plausible that DNA damage induced metaphase arrest through spindle assembly checkpoint activation (Kerr et al., 2012). There is much less known about whether hAFMSCs recognize OPA by resisting DNA damage. In this study, to determine whether hAFMSCs improved ovarian function in an OPA mouse model by inhibiting DNA damage, relevant markers were tested. Our results revealed increases in the expression of DNA damage markers to a normal level following hAFMSC therapy (**Figure 7**).

Previous study indicated that human umbilical cord mesenchymal stem cells improve the ovarian function by paracrine mechanism (Li et al., 2017). Recently, study results have demonstrated that EGF released from human placental mesenchymal stem cells could restore ovarian function through regulating oxidative stress pathway (NRF/HO-1) (Ding et al., 2020). MSC-secreted growth factors (hepatocyte growth factor and epidermal growth factor) improved oogenesis in aged mice (Ding et al., 2018). As a consequence, we found that hAFMSCs may resist DNA damage through secreting cytokines in treatment of OPA disease.

We have provided the first evidence, to our knowledge, of an interaction between hAFMSCs and OPA. The results of the present study have provided new pathogenetic insight into the view that hAFMSCs delay OPA by resisting DNA damage. Furthermore, our present study has demonstrated that hAFMSCs play a pivotal role in inhibiting ovarian aging at the ovarian development stage by reducing DNA damage. This discovery has important implications for understanding the routes of promoting the ovarian function of reproductive age through secreting growth factors (Liu et al., 2019). The new era in fertility preservation research will focus on stem cell therapy. As such, the work presented in this study and our ongoing translational work exploit a new frontier in the field of fertility preservation.

## Data Availability Statement

All datasets generated for this study are included in the article/**Supplementary Material**.

## Ethics Statement

The use of human ovarian granular cells was in accordance with the relevant guidelines and regulations, and the experimental protocols were approved by the Medical Ethics Committee of the Suzhou Hospital Affiliated to Nanjing Medical University (NJMU-2017-480). All the patients provided written informed consent prior to participation in this study. Our investigation using experimental animals was conducted on the basis of the Nanjing Medical University Animal Center's specific guidelines and standards (20170330).

## Author Contributions

BH and CD performed the cellular and molecular assays *in vivo* and *in vitro*. QZ participated in the statistical analysis and revised the manuscript. BH contributed to hGC collection and purification. QZ contributed to hGC purification culture. CD carried out the partial immunoassays. WW carried out the partial HE assays. BH and JL participated in the mice feeding. BH planned the experiments and drafted part of the manuscript. HL planned the experiments and wrote the manuscript. All the authors read and approved the final manuscript.

## Funding

This work was supported by grants from National Natural Science Foundation of China (81801515), Suzhou science and technology for people's livelihood (SYS2018081), the Suzhou expert team of clinical medicine (SZYJTD201708), and the Suzhou talent training program (GSWS2019005).

## Conflict of Interest

The authors declare that the research was conducted in the absence of any commercial or financial relationships that could be construed as a potential conflict of interest.

## Supplementary Material

Supplementary Figure 1 The survival time of hAFMSCs in OPA ovary was tested. The survival time was detected after hAFMSCs transplantation into OPA ovary at 24h, 7 days, and 14 days.

Supplementary Table 1 Designations, sequences, and the sizes of real-time PCR amplicons.

Supplementary Table 2 Information regarding the flow cytometry antibodies.

Supplementary Table 3 Information regarding western blot analysis.

## Abbreviations

OPA, Ovarian physiologic aging; hAFMSCs, Human amniotic fluid mesenchymal stem cells; hGCs, Human granulosa cells; CTX, Cyclophosphamide; HE, Hematoxylin and eosin; MSH4, MutS homolog 4; AMH, Anti-mullerian hormone; FSHR, Follicle-stimulating hormone receptor; FOXL2, Forkhead box L2; CYP19A1, Cytochrome P450 family 19 subfamily A member 1; STAG3, Stromal antigen 3; GDF9, growth differentiation factor 9; BMP15, Bone morphogenetic protein 15; γH2AX, Gamma histone variant H2AX; BRCA1, Breast cancer 1; PARP1, Poly (ADP-ribose) polymerase 1; XRCC6, X-ray repair cross complementing 6; FACS, Flow cytometry; E2, Estradiol; ELISA, Enzyme-linked immunosorbent assay; DAPI, 4', 6-diamidino-2-phenylindole; BSA, Bovine serum albumin; PFA, Paraformaldehyde; CM, Conditioned media; PGC, Primordial germ cells.
