# Supplementary figures and images for: Human Amniotic Fluid Mesenchymal Stem Cells Improve Ovarian Function During Physiological Aging by Resisting DNA Damage
Source: Front Pharmacol. 2020 Mar 26;11:272. doi: 10.3389/fphar.2020.00272 (PMC7113373; doi:10.3389/fphar.2020.00272)

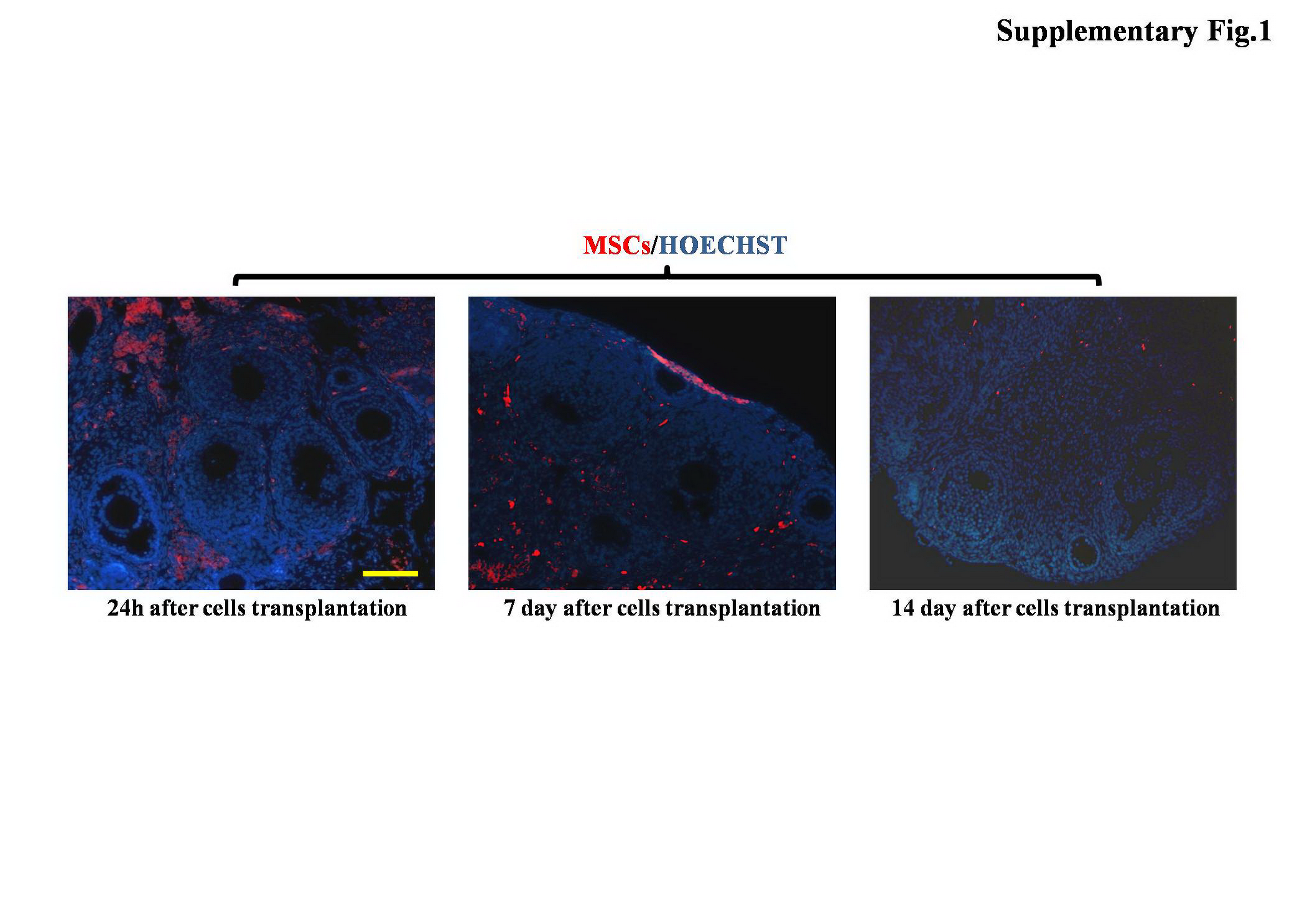

Supplement: Supplementary Figure 1 — The survival time of hAFMSCs in OPA ovary was tested. The survival time was detected after hAFMSCs transplantation into OPA ovary at 24h, 7 days, and 14 days. [file Image_1.tif]
