# Supplementary material for: Human Amniotic Fluid Mesenchymal Stem Cells Improve Ovarian Function During Physiological Aging by Resisting DNA Damage
Source: Front Pharmacol. 2020 Mar 26;11:272. doi: 10.3389/fphar.2020.00272 (PMC7113373; doi:10.3389/fphar.2020.00272)
Supplement: Supplementary Table 1 — Designations, sequences, and the sizes of real-time PCR amplicons. [file DataSheet_1.doc]

**Supplemental Table 1 Designations, sequences, and the sizes of real-time PCR amplicons**

**H=Human; M=Mouse.**

| **Name** | **Sequence from 5'-3'** | **Size (bp)** |
| --- | --- | --- |
| BRCA1 (H) Fw | AATGCTGATCCCCTGTGTGA | 70 |
| BRCA1 (H) Rev | CTCTAGGATTCTCTGAGCATGGC |
| XRCC6 (H) Fw | TTGGCTTTCGTCAGGGACAT | 119 |
| XRCC6 (H) Rev | CCCTGTACTCGGTCCCAATC |
| PARP1 (H) Fw | AACTGTATTCAGCGGCGACA | 82 |
| PARP1 (H) Rev | ACTCTTCAGTGTGCGGAAGG |
| γH2AX (H) Fw | TTGATTGCCGGGCTTAGAGG | 223 |
| γH2AX (H) Rev | CTGCGGCAGGTATAGAACTC |
| FOXL2 (H) Fw | AAATCTGCCGGTACTCGCTC | 142 |
| FOXL2 (H) Rev | GATTCTGAAAGCCGCGAGGA |
| MSH4 (H) Fw | CTGGAGGGGTCGCTCAGAA | 226 |
| MSH4 (H) Rev | TGACGTGCCAGGACAGG |
| CYP19A1 (H) Fw | TGCTGATTTCTCAGGACCGC | 160 |
| CYP19A1 (H) Rev | CTGGGATCAGCACCACTGTT |
| AMH (H) Fw | GAAGACTTGGACTGGCCTCC | 192 |
| AMH (H) Rev | GTTGCAGACCCCGAAGGTG |
| GDF9 (H) Fw | ATCAGCTCGCACACTTCCAA | 93 |
| GDF9 (H) Rev | GGGAACGTACAAGACACGCT |
| STAG3 (H) Fw | ATCAGCTCGCACACTTCCAA | 93 |
| STAG3 (H)Rev | GGGAACGTACAAGACACGCT |
| FSHR (H) Fw | CAGAGAAGCACCATGGTTTCA | 94 |
| FSHR (H) Rev | GCCTATCCACACTGACGCAT |
| BMP15 (H) Fw | CGATGGTCTCAATTCCCCCA | 214 |
| BMP15 (H) Rev | GATCTAGCCGTACTGTTGCT |
| GAPDH (H) Fw | GAAGGTCGGAGTCAACGGATTT | 223 |
| GAPDH (H) Rev | CTGGAAGATGGTGATGGGATTTC |
| BRCA1 (M) Fw | ATGGGCATGTCGTGAGTTGT | 194 |
| BRCA1 (M) Rev | TCTGGGTGTTGTTTGGTGCT |
| XRCC6 (M) Fw | GTCCTGAAGCACCTGAGTCC | 241 |
| XRCC6 (M) Rev | ATATCAGCGAGAAGCCCTGC |
| PARP1 (M) Fw | GACGCCGAGGCGATTCAAA | 100 |
| PARP1 (M) Fw | CAGCCATCCTCTCGTCCAGT |
| γH2AX (M) Fw | TTGATTGCCGGGCTTAGAGG | 223 |
| γH2AX (M) Fw | CTGCGGCAGGTATAGAACTC |
| FOXL2 (M) Fw | CAGCTTACCTCTTGGCCCTC | 88 |
| FOXL2 (M) Fw | TCTAGTCGCAGACAGTCGGG |
| MSH4 (M) Fw | TCAAATAGATGGCTCGCCCG | 114 |
| MSH4 (M) Fw | GCTCTGGCAGCAGAGTTACA |
| CYP19A1 (M) Fw | TTAGGCCAGTGAGGAACTGAC | 142 |
| CYP19A1 (M) Fw | CGGCACACTTAAGGCTGGAT |
| AMH (M) Fw | CTGGTCAACCTGTCAGACCC | 106 |
| AMH (M) Fw | GCATAGGTTCCCTCTCCGTG |
| GDF9 (M) Fw | TGGGTGGGGCTTATAGAGGT | 186 |
| GDF9 (M) Rev | CTGCAGCTTAGGGGTCTCAC |
| STAG3 (M) Fw | TTCTTCACAGTTTTCCACCCTAA | 167 |
| STAG3 (M)Rev | TGGAGCAAGGGAGCAACATT |
| FSHR (M) Fw | CTTCTTTCTGGAGAGAGGGCT | 99 |
| FSHR (M) Rev | CCCTGACCTATCTGCCATGC |
| BMP15 (M) Fw | CGACCCTACATTGCCCTCAA | 183 |
| BMP15 (M) Rev | GGCTTTACCAGCCTCACCAT |
| GAPDH (M) Fw | TTCCAGTATGACTCTACCCACGGCA | 137 |
| GAPDH (M) Rev | GCACCAGCATCACCCCATTTG |

**Supplemental Table 2. Information regarding the flow cytometry antibodies.**

| Antibody | Vender, Catalog | Country |
| --- | --- | --- |
| CD29 | Invitrogen, 12-0291-82 | USA |
| CD90 | Abcam, ab124527 | USA |
| CD73 | BD, 550257 | USA |
| CD105 | Abcam, ab18278 | USA |
| CD34 | Invitrogen, 11-0341-82 | USA |
| AMH | LSBio, LS-C319451 | USA |
| FSHR | LSBio, LS-C783210 | USA |
| FOXL2 | NOVUS, NBP2-70013 | USA |
| CYP19A1 | NSJ Bio, RQ4643 | USA |
| KI67 | BioLegend, 350504 | USA |
| Annexin V | BD, 556420 | USA |

**Supplemental Table 3. Information regarding western blot analysis.**

| Antibody | Vender, Catalog | Country |
| --- | --- | --- |
| FSHR | LSBio, LS-C783210 | USA |
| AMH | LSBio, LS-C319451 | USA |
| FOXL2 | NOVUS, NBP2-70013 | USA |
| CYP19A1 | NSJ Bio, RQ4643 | USA |
| SURVIVIN | Abcam, ab76424 | USA |
| BCL2 | Abcam, ab182858 | USA |
| CASEPASE3 | Abcam, ab32351 | USA |
| CASEPASE9 | Abcam, ab202068 | USA |
| MSH4 | NOVUS, NBP1-58170 | USA |
| STAG3 | NOVUS, NBP1-58087 | USA |
| GDF9 | Abcam, ab93892 | USA |
| BMP15 | Invitrogen, PA5-96637 | USA |
| γH2AX | Abcam, ab26350 | USA |
| BRCA1 | Sigma, SAB2702136 | USA |
| PARP1 | Abcam, ab32138 | USA |
| XRCC6 | NOVUS, NB100-1915 | USA |
| GADPH | NOVUS, NB300-221 | USA |
